# Investigation of *B-atp6-orfH79* distributing in Chinese populations of *Oryza rufipogon* and analysis of its chimeric structure

**DOI:** 10.1186/s12870-023-04082-5

**Published:** 2023-02-07

**Authors:** Xuemei Zhang, Shuying Chen, Zixian Zhao, Cunqiang Ma, Yating Liu

**Affiliations:** 1 State Key Laboratory of Conservation and Utilization of Bio-Resources in Yunnan, The Key Laboratory of Medicinal Plant Biology of Yunnan Province, Yunnan Agricultural University, Kunming, 650201 Yunnan China; 2 College of Agronomy and Biotechnology, Yunnan Agricultural University, Kunming, 650201 Yunnan China; 3 College of Horticulture, Nanjing Agricultural University, Nanjing, 210095 Jiangsu China; 4 College of Tobacco, Yunnan Agricultural University, Kunming, 650201 Yunnan China

**Keywords:** Chimeric gene, Cytoplasmic male sterility, Mitochondrial DNA, *Oryza rufipogon*, Sequence feature

## Abstract

**Background:**

The cytoplasmic male sterility (CMS) of rice is caused by chimeric mitochondrial DNA (mtDNA) that is maternally inherited in the majority of multicellular organisms. Wild rice (*Oryza rufipogon* Griff.) has been regarded as the ancestral progenitor of Asian cultivated rice (*Oryza sativa* L.). To investigate the distribution of original CMS source, and explore the origin of gametophytic CMS gene, a total of 427 individuals with seventeen representative populations of *O. rufipogon* were collected in from Dongxiang of Jiangxi Province to Sanya of Hainan Province, China, for the PCR amplification of *atp6, orfH79* and B-a*tp6-orfH79*, respectively.

**Results:**

The B-*atp6-orfH79* and its variants (B-*atp6-*GSV) were detected in five among seventeen populations (i.e. HK, GZ, PS, TL and YJ) through PCR amplification, which could be divided into three haplotypes, i.e., BH1, BH2, and BH3. The BH2 haplotype was identical to B-*atp6-orfH79,* while the BH1 and BH3 were the novel haplotypes of B-*atp6-*GSV. Combined with the high-homology sequences in GenBank, a total of eighteen haplotypes have been revealed, only with ten haplotypes in *orfH79* and its variants (GSV) that belong to three species (i.e. *O. rufipogon*, *Oryza nivara* and *Oryza sativa*). Enough haplotypes clearly demonstrated the uniform structural characteristics of the B-*atp6-orfH79* as follows: except for the conserved sequence (671 bp) composed of B-*atp6* (619 bp) and the downstream followed the B-*atp6* (52 bp, DS), and GSV sequence, a rich variable sequence (VS, 176 bp) lies between the DS and GSV with five insertion or deletion and more than 30 single nucleotide polymorphism. Maximum likelihood analysis showed that eighteen haplotypes formed three clades with high support rate. The hierarchical analysis of molecular variance (AMOVA) indicated the occurrence of variation among all populations (*F*_ST_ = 1; *P* < 0.001), which implied that the chimeric structure occurred independently. Three haplotypes (i.e., H1, H2 and H3) were detected by the primer of *orfH79*, which were identical to the GVS in B-*atp6*-GVS structure, respectively. All seventeen haplotypes of the *orfH79,* belonged to six species based on our results and the existing references. Seven existed single nucleotide polymorphism in GSV section can be translated into eleven various amino acid sequences.

**Conclusions:**

Generally, this study, indicating that *orfH79* was always accompanied by the B-*atp6*, not only provide two original CMS sources for rice breeding, but also confirm the uniform structure of B-*atp-orfH79,* which contribute to revealing the origin of rice gametophytic CMS genes, and the reason about frequent recombination of mitochondrial DNA.

**Supplementary Information:**

The online version contains supplementary material available at 10.1186/s12870-023-04082-5.

## Background

Cytoplasmic male sterility (CMS) caused by *orfH79* and its variants has been used to produce rice hybrid seeds of several types gametophytic CMS lines on a commercial scale, which eliminates the need for hand emasculation [1–4]. The CMS genes are located in the mitochondrial genome [5–12], and maternally inherited in the majority of angiosperms [13, 14]. Given its maternal inheritance, the original CMS source can be bred into the CMS line through the successive back-crossing as the female parent for the selection of different isoplasmic allonuclear male-sterile plant [15]. Plant mitochondrial DNA (mtDNA) is highly conserved as a consequence of its maternal inheritance. And its notoriously complex structure [14, 16] reflects in large part the frequent occurrence of recombination, integration, and deletion of DNA sequences [17, 18]. Almost all reported CMS genes are chimeric structure, i.e., the exogenous sequence in the gene originated from nuclear DNA, chloroplast DNA, or mtDNA itself [19]. Double-strand break repair is one hypothesized mechanism for mtDNA recombination [20]. Cell-to-cell movement of mitochondria through a stem graft junction involving two tobacco (*Nicotiana*) species has been reported [21]. If the problem of CMS is regarded as a tree, the application of the sterile gene is the crown, the action principle of the sterile gene is the trunk, while the origin of the sterile gene is the root. At present, the studies about the first two are too numerous to list, but the latter is too few. However, the mechanism by which chimeric CMS genes arose and resulted in sterility with such consistency is uncertain.

The generation of CMS system mainly attributed to the three phenomena as follows: (1) spontaneous sterility, such as rice Wild Abortive type CMS (CMS-WA) [22], Polish rape CMS-POL, and radish CMS-Ogura [23]; (2) distant hybridization, such as rice Boro II type CMS (CMS-BT) [24], HongLian type CMS (CMS-HL) [22], and sorghum CMS-IS1112C (A3) [25]; and (3) somatic hybridization [26]. Among them, distant hybridization is the most common means of generating CMS system. For instance, distant hybridization is responsible for more than sixty CMS original sources from rice in China [27], and eighty-four CMS original sources from corn in the USA [28]. Additionally, the interspecific hybridization also generated seventy-two CMS sources in sunflower [29]. Transgene can also produce male sterility, which have not been applied in the production [30].

Generally, some CMS systems in crops are considered to have originated in their closely related wild species with sterility gene. A survey of the CMS gene *orfH522* distribution in sunflower, using a gene-specific primer among more than 1,200 plants representing fifty-five accessions of *Helianthus annuus* and twenty-six accessions of *Helianthus petiolaris*, failed to detect CMS cytotypes in natural populations [31]. The *orf138* gene responsible for *Ogura* CMS, arose only once in the evolution of radish as indicated by a survey of wild and cultivated radish accessions that were grown or distributed where the *Ogura cytoplasm* was originally identified in Japan [32]. The detection of *orf138* gene in natural populations of *Raphanus raphanistrum* remained at a low frequency in Europe, and a variant of *orf138* coding sequence has lost its ability to induce male sterility [33]. Li et al. [34] detected seven *orfH79* haplotypes in accessions of four *Oryza* species (i.e. *Oryza meridionalis*, *Oryza nivara*, *Oryza barthii* and *Oryza rufipogon*) among forty-two accessions of five wild rice species collected by the International Rice Research Institute (IRRI). The rice CMS gene, *orf352,* is also carried by *O. rufipogon* with several amino acid haplotypes [35]. He et al. [36] has analyzed the sequence including the B*-atp6-orf79* like structure and the *atp6* in the GenBank database.

Wild rice (*O. rufipogon* Griff.) is the closest wild relative of Asian cultivated rice (*Oryza sativa* L.) and has been considered as an ancestral progenitor of the *O. sativa* [37–40]. China is an important center of diversity in the extensive distribution of *O. rufipogon*. The latitudinal range between 18°17′ and 28°05′ N is the most abundant distribution area of *O. rufipogon* in China, and the resident populations formerly harbored luxuriant genetic diversity [41, 42]. For example, the CMS-HL cytoplasm resource is derived from *O. rufipogon* with a red awn distributed on Hainan Island, China [22].

The genes *orf79*, *orfH79*, and *L-orf79* are responsible for CMS-BT [1, 4, 12, 43], CMS-HL [3, 8], and CMS-Lead (Lead type CMS) [2], respectively. Particularly, *L-orf79* gene is also detected in the CMS-Liao (Liao type CMS) cytoplasm type, but it is not identified as its sterility gene [44]. *L-orf79* gene sequence have no difference in sequence between CMS-Lead and CMS-Liao lines. The *orfH79* gene and its variants (hereafter abbreviated as GSV for convenience) are adjacent to and co-transcribed with B-*atp6,* and it’s a 52 bp downstream connected by an abundant variance 176 bp sequence, which thus constitute the B-*atp6-orfH79* region. B-*atp6-orfH79* and its variants (hereafter abbreviated as B-*atp6*-GSV) is transcribed as a single B-*atp6*-GSV RNA molecule [45–48]. The sequence structure analysis of B-*atp6*-GSV would provide an improved understanding the origin of CMS gene, and the molecular mechanism of mtDNA recombination.

The mechanisms of mtDNA recombination provide insight into the causes of its complex structure [49]. In addition to the important application for hybrid seed production, CMS also can be used to research mtDNA transfer [50], the interaction of nuclear DNA and mtDNA, the origin and maintenance of stable gynodioecy in plants [29, 51, 52], and floral organ development [21, 53]. In this study, we surveyed the CMS genetic differentiation among seventeen populations of *O. rufipogon* across its entire distribution range in China. Gene-specific primers were used to amplify B*-atp6-orfH79*, *orfH79* and *atp6* through polymerase chain reaction (PCR), and their sequences were analyzed to address the following aims: (1) to determine if a natural population in China carries the gametophytic CMS gene or variants and, if so, to explore its evolutionary relationships among different haplotypes; (2) to determine the distribution of haplotypes among populations and identify additional cytoplasm resources for rice breeding; (3) to analyze the sequence structure characteristics and uniformity of the chimeric trait of B*-atp6-orfH79*.

## Results

### Detection of atp6, B-atp6-orfH79 and orfH79 in Oryza rufipogon

In this study, each individual in all populations can be amplified products using the primers for the *atp6* gene (Table 1), indicating the wide presence of *atp6* gene in *O. rufipogon* populations, which was consistent with the previous study that mtDNA is conservative [19]. To verify whether *orfH79* and its variants (GSV) were always accompanied by B*-atp6*, the specific primers for *orfH79* and B*-atp6-orfH79* were employed. Both primer pairs amplified products for each individual of the HK, GZ, PS, TL, and YJ populations (Table 1), which indicated that the GSV were always accompanied with B*-atp6.* Conversely, PCR products were not amplified with both primer pairs for the remaining twelve populations, i.e., NHNC, WN, QH, NN, LB, XZ, HZ, GP, YL, FC, BH and DX. The detection of *orfH79* gene from the five populations of *O. rufipogon* in China at a medium frequency about 30%, provided abundant potential CMS sources for rice breeding.

**Table 1:** Detailed information about 17 populations of *Oryza rufipogon* collected from China including geographical location, altitude range, longitude, latitude and ecotype

Population localities	Population code	Species	Number	Altitude (m a.s.l.)	Longitude (E)	Latitude (N)	Ecotype
Hainan, Sanya	NHNC	*Oryza rufipogon*	15	3	109°29′	18°17′	stolon
Hainan, Wanning, Dongao	WN	*Oryza rufipogon*	25	45	110°.37	18°.73	stolon
Hainan, Qionghai, Zhongyuan	QH	*Oryza rufipogon*	25	56	110°29′	19°06′	erect
Hainan, Haikou, Longhua	HK^*^	*Oryza rufipogon*	25	92	110°20′	19°57′	Head-up tilt
Yunnan, Yuanjiang, Donger	YJ	*Oryza rufipogon*	5	700	102°	23°59′	erect
Guangxi, Longan, Natong	NN	*Oryza rufipogon*	20	98	108°12′	22°40′	erect
Guangxi, Laibin, Shiya	LB	*Oryza rufipogon*	30	133	109°27′	23°28′	Head-up tilt
Guangxi, Xiangzhou, Yunjiang	XZ	*Oryza rufipogon*	30	336	109°46′	24°07′	Stolon/erect
Guangxi, Zhongshan, Chengxiang	HZ	*Oryza rufipogon*	30	438	111°19′	24°34′	Head-up tilt
Guangxi, Guiping, Xunwan	GP	*Oryza rufipogon*	30	113	110°08′	23°24′	stolon
Guangxi, Yulin, Fumian	YL	*Oryza rufipogon*	30	237	110°04′	22°23′	Head-up tilt
Guangxi, Fancheng, Huashi	FC	*Oryza rufipogon*	30	71	108°11′	21°45′	stolon
Guangxi, Beihai, Fucheng	BH	*Oryza rufipogon*	27	54	109°18′	21°35′	Stolon/ head-up tilt
Guangdong, Gaozhou, Zhenjiang	GZ	*Oryza rufipogon*	30	91	110°42′	21°51′	stolon
Guangdong, Gaozhou, Zhenjiang	PS	*Oryza rufipogon*	25	-57	110°42′	21°48′	head-up tilt/ stolon
Guangdong, Gaozhou, Zhenjiang	TL	*Oryza rufipogon*	26	83	110°44′	21°47′	head-up tilt/ stolon
Jiangxi, Dongxiang, Gangshangji	DX	*Oryza rufipogon*	24	276	116°31′	28°05′	Head-up tilt

^*^, indicate that this population has disappeared since 2015

As conserved gene sequence, no differences were found among all the sequences of *atp6* in the *O. rufipogon* populations. Fifteen individuals were randomly selected for sequencing from each population, except for the YJ population with only five individuals. The sequencing results revealed that only one haplotype was identified in all individuals of one population.

**Fig. 1:**
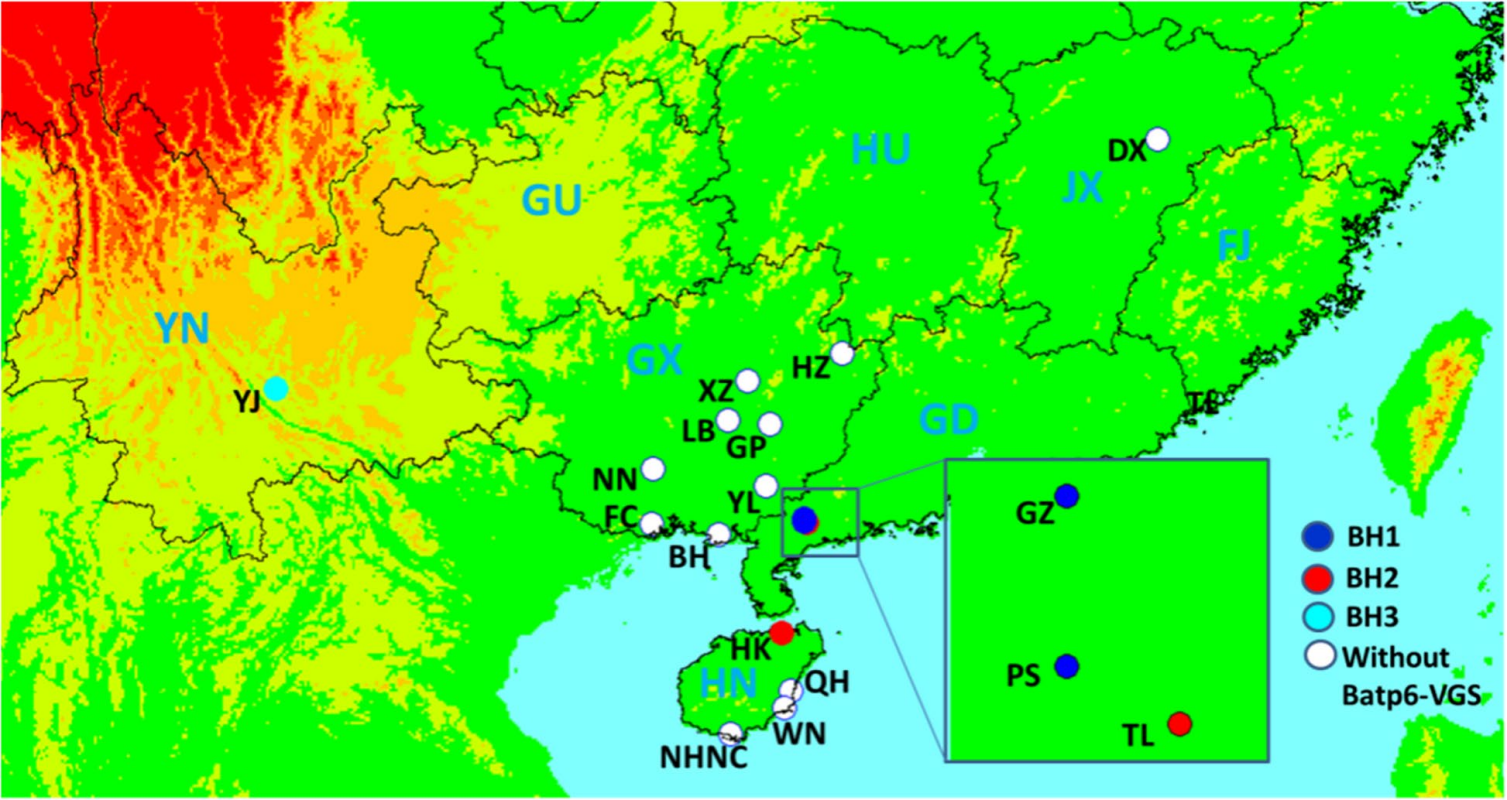
Collection localities in China, and the distributions of the three mitochondrial haplotypes in seventeen *Oryza rufipogon* populations

Among five populations, three haplotypes (named as H1, H2, and H3, respectively) were detected with the primers for *orfH79*, and three haplotypes (named as BH1, BH2, and BH3, respectively) were detected with the primers for B*-atp6-orfH79*. Specifically, The H1, H2, and H3 sequences were identical to the corresponding sequences of BH1, BH2, and BH3 in each population, respectively (Table 2, Table 3 and Fig. 1). For instances, the PS and GZ populations shared H1 and BH1, while the TL and HK populations shared H2 and BH2. Furthermore, H3 and BH3 were only detected in the YJ population.

**Table 2:** Primers used in this study and PCR products amount in each relevant population

Primer name	Primer sequence	PCR products amount
*orf79*	F: 5’- ATGACAAATCTGCTCCGATG -3’	GZ(30),HK(30),YJ(5), TL(25),PS(26)
	R: 5’- CTTACTTAGGAAAGACTAC -3’	
B-a*tp6-orfH79*	F: 5'-TCCTTGTCTATGGCGGTAA-3'	GZ(30),HK(30),YJ(5), TL(25),PS(26)
	R: 5'-GAGCAAACCACCACTGTCC-3'	
*atp6*	F: 5'-CTGAATGGAGGAACGGCGAT-3'	Every individual
	R: 5'-AGCATAGTCCAAGCGAACCC-3'	

*F* forward primer, *R* reverse primer. Populations and the numbers that could be detected amplified products

**Table 3:** *OrfH79* and its variants (GSV), and B-*atp6-orfH79* and its variants (B-*atp6-GSV*) among the populations of *Oryza rufipogon* in China

DNA section			
Haplotype	Population (n)	Haplotype	Population (n)
H1	PS16(15), GZ30(15)	BH1	PS16(15), GZ15(15)
H2	TL(15), HK(15)	BH2	TL22(15), HK6(15)
H3	YJ(5)	BH3	YJ5(5)

The number of individuals sequenced in each population in the brackets

### Sequence structure characteristics of B-atp6-GSV, phylogenetic analysis and the uniform chimeric trait

**Fig. 2:**
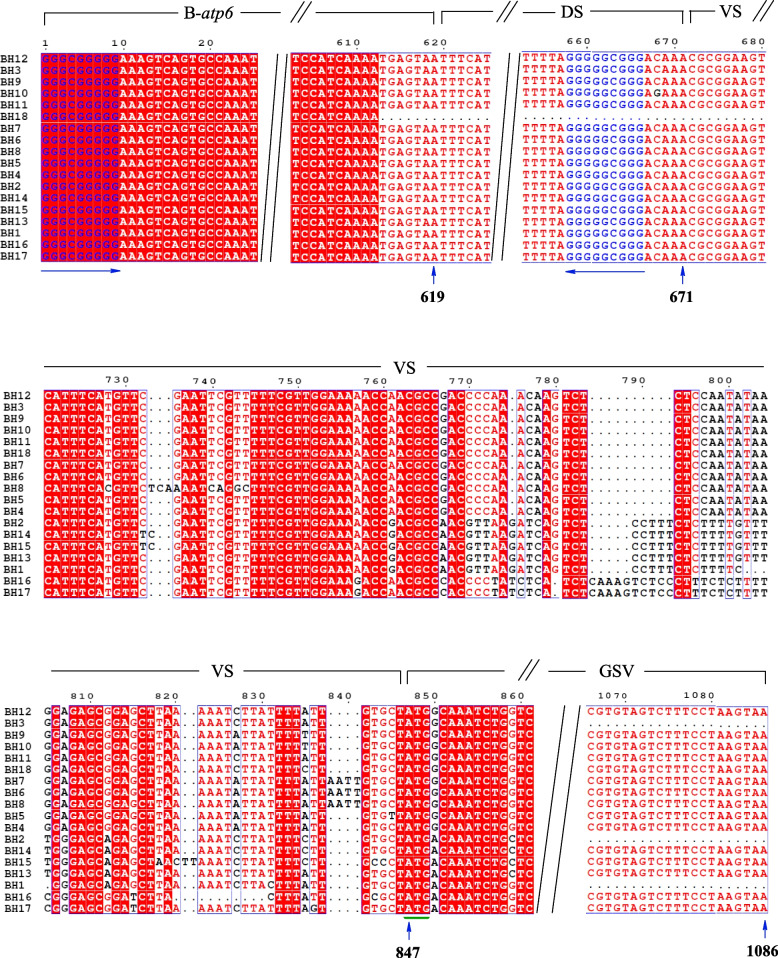
The sequence structure characteristics of mitochondrion B-*atp6-orfH79* and its variants (B-*atp6-*GSV) (i.e. from BH1 to BH18) of *Oryza* in sampled population from China and collected from GenBank

B-*atp6-orf79*, B*-atp6-orfH79*, and B*-atp6-L-orf79* are previously published haplotypes in *O. rufipogon* [1–4]. Among three detected haplotypes in this research, BH2 was identical to B*-atp6-orfH79*, whereas BH1 and BH3 were novel haplotypes in *O. rufipogon* populations. B*-atp6-orf79*, B*-atp6-orfH79*, B*-atp6-L-orf79*, BH1, and BH3 constitute the B-*atp6*-GSV region. Previously, B-*atp6-orf79* has been detected in the CMS-Dian 1 and CMS-BT cytoplasm types, and B*-atp6-L-orf79* was detected in the CMS-Lead and CMS-Liao types [4, 44]. A total of eighteen haplotypes (i.e. BH1-BH18) were summarized from the GenBank, and their common characteristics and unique variation were demonstrated in Fig. 2. Their accession number in the GenBank of NCBI database, and other information such as population and species have been provided in Additional file 1: Table S1. All were chimeric sequences, containing a 671 bp conserved sequence composed of B-*atp6* (619 bp) and 52 bp downstream of the B-*atp6* (DS). B-*atp6* and DS was identical to the corresponding sequences of *atp6* and its corresponding downstream of *O. sativa* and *O. rufipogon* [54]. There were no differences in sequence among these eighteen haplotypes, except for BH18 and a single nucleotide polymorphism in 668 position (C turned into G) of BH10. The complex variable sequence (VS) connected with the DS and GSV, is a 176 bp sequence including five insertion or deletion and more than 30 single nucleotide polymorphisms, as shown in Fig. 2.

**Fig. 3:**
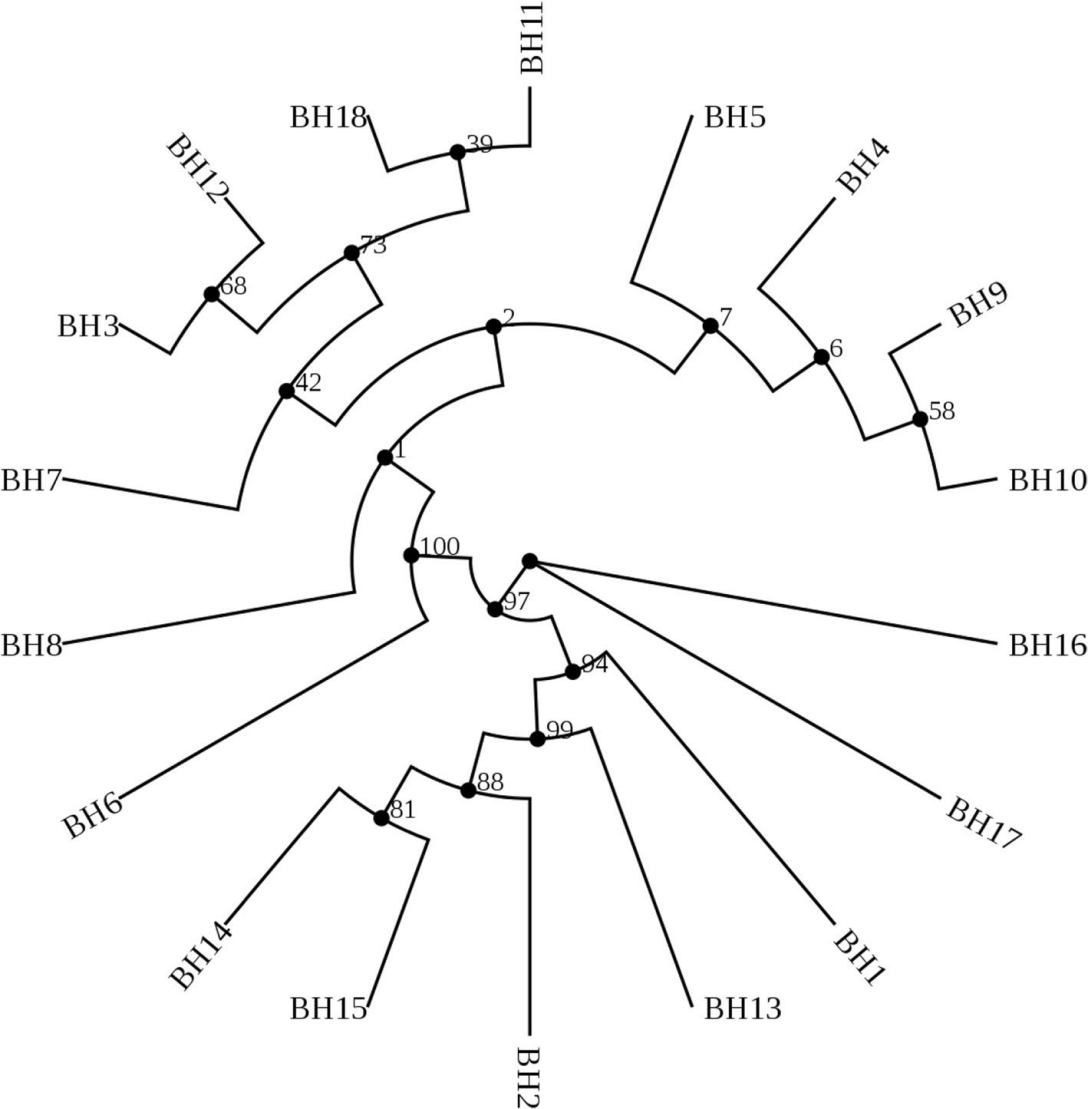
Maximum likelihood tree constructed by model HKY85 using eighteen haplotypes of B-*atp6*-*orfH79* from three species of *Oryza* (the 5’ last 26 bp of the *orfH79* was not used in analysis)

Maximum-Likelihood Phylogenies analysis of B-*atp6*-GSV of eighteen haplotypes (Fig. 3) indicated that three clades are formed with a high support rate. Owing to primer selection, the last 26 bases of GSV were not amplified. Thus these 26 bases were not included in the present and subsequent analysis. BH16 and BH17 formed a clade, while BH1, BH2 and BH13-15 formed another clade. Additionally, the other haplotypes (i.e. BH3-BH12, BH18) formed the third clade (Fig. 3). The new haplotype BH1 was close to the BH13, while another new haplotype BH3 and the existing BH12 clustered together as a sub-clade. BH16 and BH17 formed a clade with no sequential relationship, and both only exist in *O. rufipogon* distributing in south Asia (only 4 time were detected). Specifically, BH17 was only detected in Thailand, while BH16 was detected in India and Sri Lanka [36]. Additionally, the BH1-2 and BH13-15 formed another clade with a high support rate of each branch, which all were only detected in *O. rufipogon*. As a new haplotype, BH1 is located on Chinese mainland such as Guangdong Province, while the BH2 haplotype both distribute in Chinese mainland and Hainan Island, including Zhenjiang Town of Guangdong Province and Longhua District of Hainan Province (Fig. 1). Generally, the BH2, located in the south of BH1 in the distribution of populations, also was reported in Thailand. The BH13 was located in China, while both BH14 and BH15 are located in India [36]. However, the WN and QH population close to the BH13 location, did not carry the *B-atp6-*GSV, indicating its independent occurrence*.*

The third clade has a support rate of 100% that all the haplotypes from *O. sativa* gather in these branches, while the support rate is relatively low inside the clade. The BH6 and BH8, located in India (They were detected once, respectively), were only carried by *O. rufipogon*, and BH4-5 and BH9-10 are grouped into one branch and carried by *O. rufipogon*, *O. sativa* or *O. bathii*, respectively. The BH4 haplotype could be found in all these three *Oryza* species, while BH5 and BH9 haplotypes were carried by both *O. rufipogon* and *O. sativa.* However, the BH10 haplotype only could be found in *O. sativa* grown in Australia. Except for the BH6, the other haplotypes detected in *O. sativa* are located in this third-level clade. All the haplotypes carried by Australia *O. sativa* distribute in this clade. For BH5, the sources of *O. sativa* are very wide. The *O. rufipogon* populations distributing in India, Bangladesh and Thailand carried BH4, BH5, BH9 haplotypes, respectively. The BH7 haplotype was found in *O. rufipogon* populations from India, and the *O. sativa* populations from Philippines and Africa (Nigeria, Madagascar) [36]. The BH11-12 haplotypes were only detected in China. Apart from one material from Thailan, the BH18 haplotype was only found in the materials from China. As a novel haplotype only found in Yunnan Province, China, the forming reason of BH3 needs to be further studied. Generally, the geographical distribution of haplotypes has certain geographical characteristics. Nonetheless, the B*-atp6*-GSV does not obey the distribution of general geography. There are certain species boundaries, but not absolutely. For example, all haplotypes from *O. sativa* are clustered in the third branch, which also could be found in the wild rice.

**Fig. 4:**
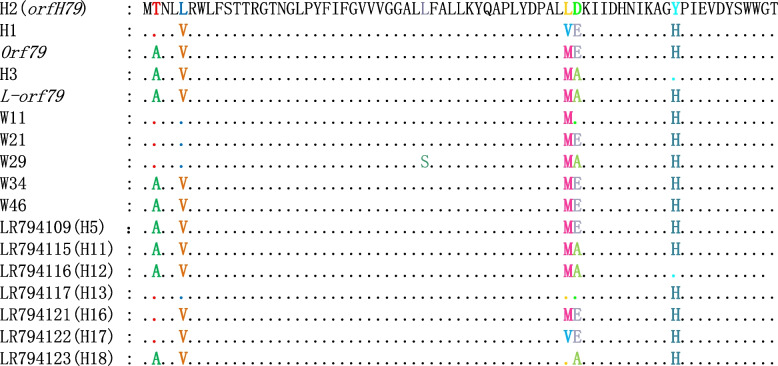
Deduced amino acid sequences of the seventeen haplotypes VGS (*orfH79* and its variants)

The populations carrying the B*-atp6*-GSV appear irregularly, have several different phenotypes (Additional file 2: Fig. S1.). The enough haplotypes of B*-atp6*-GSV provide a clear structure, including their consistency and variation. We speculated that B*-atp6* has the uniform chimeric trait in *B-atp6-*GSV structure, because of the inverted repeat sequence like GGGCGGGGG……GGGGGCGGG in the B-*atp6* structure. Li et al. [34] reported additional seven haplotypes of *orfH79*, i.e., W11, W15(34), W20, W21, W29, W34, and W46, together with the GSV section in eighteen BH haplotypes of B-*atp6*-GSV that could be divided into ten GSV haplotypes that belong to three species (i.e. *O. rufipogon*, *O. nivara* and *O. sativa*). Seventeen haplotypes have been found in the GSV section belonging to six species of *Oryza* (Table 4)*,* due to the completely identical sequences, which demonstrated the inconformity to the classification results of B-*atp6*-GSV. Concretely, BH4-BH6, BH8-BH10 have the same GSV sequence to W46 and the *orf79*, while BH2, BH14, BH15 have the same GSV sequence to W42, W45, YtA, and the *orfH79.* Additionally, BH7 is the same as the L-*orf79*. All these seventeen GSV haplotypes can be translated into eleven different amino acid sequences as the variation of W34, W46, LR794109 and *orf79* codes for the same amino acid sequence (Fig. 4). Haplotype W20 was identical to W15 and W42 except for the variation in the 26 bases. The first 34 bases of GSV, identical to the corresponding sequence of cytochrome oxidase subunit II (COXII), was shared with nuclear DNA, which indicated that all GSV are chimeric sequences.

**Table 4:** The identity between different haplotypes

Haplotype	Haplotype	Identity (%)	Haplotype	Haplotype	Identity (%)
BH1	BH2-HL	99%	BH2-HL	BH3	98%
BH1	BH3	98%	BH2-HL	BT-Dian 1	97%
BH1	BT-Dian 1	98%	BH2-HL	Lead-Liao	97%
BH1	Lead-Liao	97%	BH3	BT-Dian 1	100%
BT-Dian 1	Lead-Liao	100%	BH3	Lead-Liao	99%

BH2-HL, indicates BH2 haplotype and B*-atp6-orfH79*, they are identical; BT-Dian 1, indicates B-*atp6-orf79*, it was detected in the CMS sterile line of *CMS-BT* and *CMS Dian 1*; *Lead-Liao*, indicates the haplotype of B-a*tp6-L-orf79*,this haplotype was detected in *CMS-Liao* and *CMS-Lead* sterile line

### Population differentiation

The hierarchical analysis of molecular variance (AMOVA) indicated the occurrence of variation among all populations (*F*_ST_ = 1; all partitions were significant at *P* < 0.001). According to the VS region of B-*atp6*-GSV (Fig. 2), the YJ population was formed as group 1, while other populations including the HK, GZ, PS, and TL populations, comprised as group 2. Of the total variation, 33.3% was partitioned among groups and 66.7% among populations within the groups (all partitions were significant at *P* < 0.001) (Table 5). Furthermore, there was no variation found within each population.

**Table 5:** Variable nucleotide sites in gametophytic male sterility *orfH79* gene and its variance (GSV)

Haplotype or accession number	No	Nucleotide Site								
		4	1 3	6 7	9 5	1 4 2	1 4 6	1 4 7	1 7 8	2 2 6
H2		A	C	T	T	T	A	C	T	?
H1			G			G		A	C	
H3		G	G			A	C	T		
orfH79										G
orf79		G	G			A		A	C	G
L-orf79		G	G			A	C	A	C	G
YtA										G
W11						A			C	G
W15										G
W20										A
W21						A		A	C	G
W29					C	A	C	T	C	G
W34		G	G	C		A		A	C	G
W42										G
W46		G	G			A		A	C	G
LR794116 (H12)		G	G			A	C	T		G
LR794113 (H9)		G	G			A		A	C	G
LR794114 (H10)		G	G			A		A	C	G
LR794110 (H8)		G	G			A		A	C	G
LR794112 (H5)		G	G			A		A	C	G
LR794109 (H5)		G	G			A		A	C	G
LR794115 (H11)		G	G			A	C	T	C	G
LR794123 (H18)		G	G			C	C	T	C	G
LR794111 (H7)		G	G			A	C	A	C	G
LR794108 (H4)		G	G			A		A	C	G
LR794119 (H14)										G
LR794120 (H15)										G
LR794118 (H2)										G
LR794117 (H13)									C	G
LR794121 (H16)			G			A		A	C	G
LR794122 (H17)			G			G		A	C	G

., indicates that the character states are the same as H2 haplotype;?, Indicate the character states are unknown. Haolotypes YtA, W11, W15, W20, W21, W29, W34, W42, and W46 have been described in the reference of Li et al., 2008. L-orf79 has been described in the reference of Kazama et al.2016, while orf79 described in the report of Wang et al.2006. LR794116- LR794122 were the sequences of B*-atp6-orfH79* in the reference of He et al. 2020 and their GSV sections were renamed as H2, H3-H18, according to the above mentioned method in this study. The same base plate color indicates the same haplotype

### Phylogenetic reconstruction for GSV

**Fig. 5:**
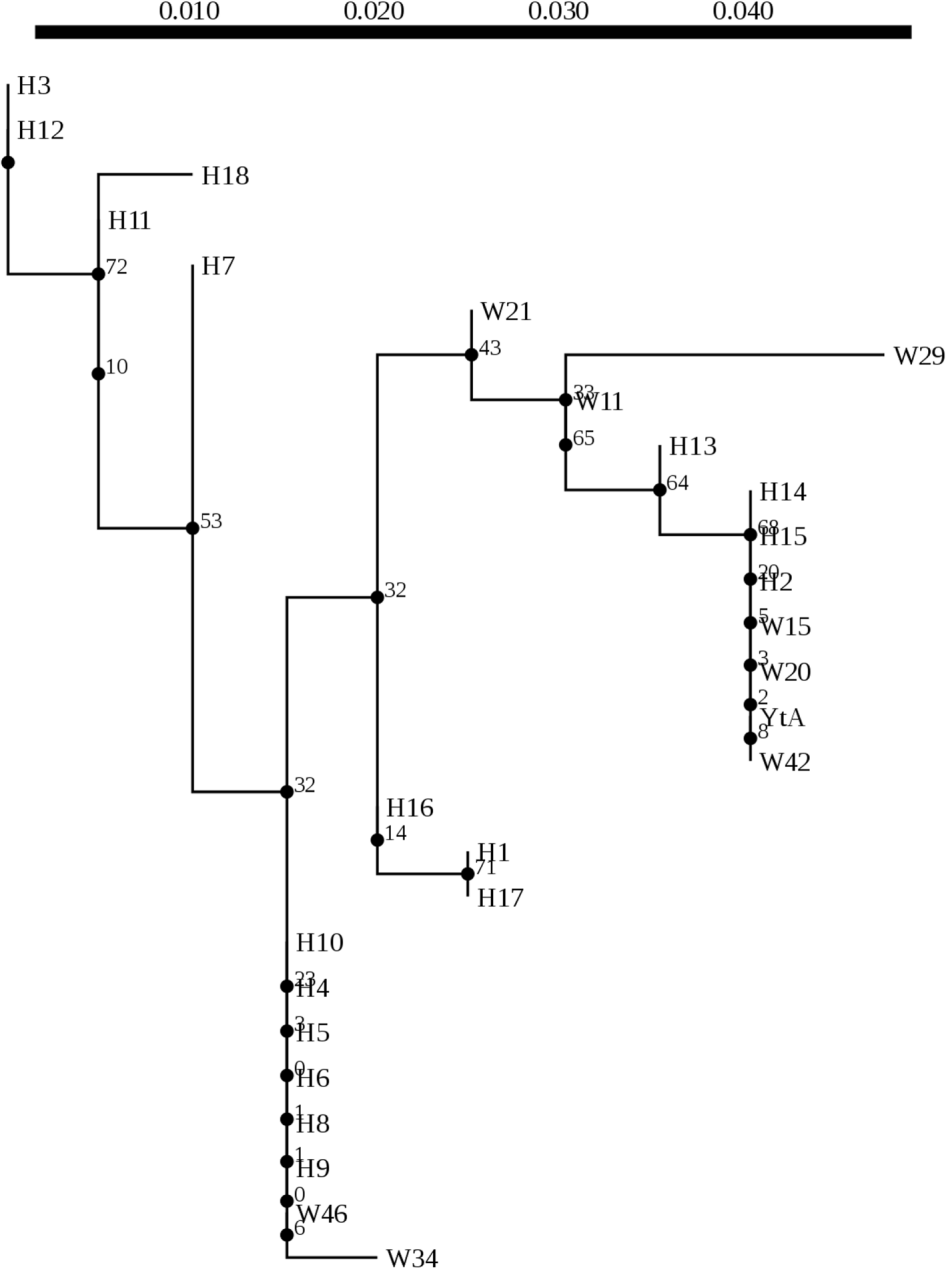
Maximum likelihood tree constructed by model HKY85 using seventeen VGS (*orfH79* and its variants) of from 6 species of *Oryza* (the 5’ last 26 bp was not used in analysis)

In this study, three GSV haplotypes including H1, H2 (*orfH79*) and H3 were detected in *O. rufipogon*. In addition, *orf79* and L*-orf79* are well-known gametophytic CMS genes. For example, *orf79* is the sterility gene of CMS-BT and CMS-Dian1 (Dian-1 type CMS), while L*-orf79* is the sterility gene of CMS-Lead. Furthermore, W11, W21, W29, W34, W42, and W46 are different variants of *orfH79* in the wild rice [34]. As shown in Fig. 5, the maximum likelihood (ML) analysis could not reflect the genetic relationship among six different species according to their comprehensive character. The TL population carried the H2 (*orfH79*) haplotype, its average kernel set just 4.6%, and the seed setting rate of 73.08% individual was 0 (Additional File 3: Table S2). The PS and GZ population carried the H1 haplotype, and their average kernel were 33.6% and 19.2%, respectively. Corresponding amino acids of variable nucleotide sites for each haplotype of GSV are shown in Fig. 4, and the variable nucleotide sites are shown in Table 4. Overall, single nucleotide polymorphisms of 7 loci bring eleven different amino acid sequences.

## Discussion

### Gametophytic CMS genes and variants may be applied as novel original CMS sources

The BH2 haplotype, detected in the HK and TL populations (red circles in Fig. 1), was identical to B*-atp6-orfH79,* which is consistent with the previous finding that the CMS-HL source originated from the distant hybridization between wild rice (*O. rufipogon* from Hainan Province, China) and *O. sativa* subsp. *indica* ‘Liantangzao’ [22]. The HK population grown in Hainan Island, China with erect and stout stem, harbors the BH2 haplotype, that do not flower in October. However, despite the identical haplotype, the TL population in Guangdong Province, China could produce thin stolons and flowers in October. These populations can serve as the novel original CMS sources. Until now, L*-orf79*, *orfH79*, and *orf79* have been considered as gametophytic CMS genes. Each variant haplotype of *orfH79* represents a novel CMS type [34], which suggested that expression of the gametophytic CMS genes and the pollen-sterility phenotype resulted from the loss of relevant restorer-of-fertility genes during the distant hybridization. PS, GZ, and YJ populations carrying BH1 and BH3 haplotypes can provide novel original CMS sources for further investigation. A random survey of TL, PS and GZ populations all located in Guangdong province, indicated that the seed setting rate varied significantly (Additional file 3: Table S2, Table S3 and Table S4). For example, the kernel set in TL population was just 4.6%, while the kernel set in PS population reached 33.6%, which should be attributed to the negative impact of environment, climate and temperature on pollen development, or pollen sterility caused by the gametophytic sterility gene.

### Geological and ecological factors may contribute to the unique haplotype

Generally, the distribution of haplotypes has certain geographical characteristics. However, the B-*atp6*-GSV does not completely obey the distribution of general geography. For examples, the first clade distribute in south Asia. However, the haplotypes of the second clade found in China and India (BH1-2 and BH13 from China, but BH14-15 from India), respectively, indicated their certain and limited geographical characteristics. In this study, the HK population is located in the north of Hainan Island, China, whereas the TL population is situated in southern continental China. Although these two populations are separated by the South China Sea and Leizhou Peninsula (Fig. 1), both populations harbored the BH2 haplotype. Despite the close geographic proximity among the GZ, TL and PS populations with a mutual linear distance below 10.0 km (Table 6 and Fig. 1), the GZ and PS populations shared the BH1 haplotype, whereas the TL population harbored BH2 haplotype. Additionally, the BH2 was also detected in the north of the PS population. The above phenomenons and results indicated the genetic recombination among various populations independently. The high support rate of the ML tree of the B-*atp6-*GVS illustrated that BH1and BH2 forms different taxon with high support, respectively. Conversely, the BH3 haplotype was only detected in the YJ population of wild rice, that is located in Yuanjiang of Yunnan Province, China. All of these populations grow in transitional areas between subtropical and tropical climates [55]. We inferred that these unique haplotypes were produced because of the geological and ecological niches of the transitional area between the tropical and subtropical climatic zones. A unique haplotype coincident with geological and ecological factors has been reported previously in yews [56]. Unfortunately, the HK population is extinct with no individuals of *O. rufipogon* as a result of real estate development and duck farming, when we returned to the sampling site in January 2016, the second year of our sampling. Protection of the TL population, which also harbors B-*atp6*-orfH79, is a matter of urgency. The origin of the unique BH3 haplotype in Yuanjiang of Yunnan Province, deserves further study.

**Table 6:** Hierarchical analysis of molecular variance (AMOVA) of samples of *Oryza rufipogon* based on nucleotide sequences of Batp6-VGS

Source of variation	d.f	Sum of squares	Variance components	Percentage of variation	Fixation indices
Among group	1	3.462	0.16667Va	33.33%	F_CT_ = 0.33333
Among population	3	15	0.33333Vb	66.67%	F_SC_ = 1
Within population	60	0.000	0.00000Vc	0	
Total	64	18.462	0.5		F_ST_ = 1

### The uniform chimeric trait

Eighteen haplotypes of B-*atp6*-GSV have been identified at present (Fig. 2). Particularly, BH1 and BH3 were first distinguished in this present study, whereas B-*atp6-orf79*, B-*atp6-orfH79*, and B-*atp6-L-orf79* have been identified previously and correspond to the CMS-BT, CMS-HL, and CMS-Lead genes, respectively [43, 48]. The original CMS-BT source arose through interspecific hybridization between *O. sativa* subsp. *indica* ‘Chinsurash Boro II’ (origin in India) and *O. sativa* subsp. *japonica* ‘Taizhung 65’ [25]. The original CMS-Dian 1 source was bred from sterile plants of ‘Taibei 8’ (a *japonica* rice grown at low altitude) planted in Baoshan, Yunnan Province in 1965. The sterile individual was speculated to be a result of the spontaneous hybridization between ‘Taibei 8’ and non-glutinous rice (*O. sativa* L.) grown at high altitude [15]. *Orf79* is also considered to be associated with CMS-Dian 1 [48]. The original CMS-HL source arose through the distant hybridization between wild rice (grown at Lingshui County, Hainan Island, China) and *O. sativa* subsp. *indica* ‘Liantangzao’ [22]. The original CMS-Lead source was identified by Watanabe (1971) [57], who hybridized *O. sativa* from Myanmar, ‘Lead Rice’, and the *japonica* cultivar ‘Fujisaka 5’ [46]. The original source of CMS-Liao line has been developed using the cytoplasm of ‘IR24’ [58].

Among the aforementioned plant material, ‘Chinsurash Boro II’ (planted in India), ‘Taibei 8’ (planted in Taiwan, China), ‘Lead Rice’ (planted in Myanmar), ‘IR24’, and the YJ, HK, TL, PS, and GZ populations all harbor B-*atp6*-GSV with wild distribution and geographical separation. Based on the different haplotypes of B-*atp6*-GSV and their distribution and the phenotypes of *O. rufipogon* populations*,* and the ML analysis, we speculated that the different haplotypes of B-*atp6*-GSV did not show a single, common origin and the chimerism of B-*atp6*-GSV occurs independently. The sequence structure of B-*atp6*-GSV includes a conserved sequence 5′-GGGCGGGGG……GGGGGCGGGACAAA-3′ (671 bp), of which GGGCGGGG……TGAGTAA (B*-atp6*, 610 bp) identical to the corresponding sequence of rice *atp6*, and TTTCATAA……GGGGGCGGGA (52 bp) that is a downstream of the rice *atp6* (Fig. 2) [54]. The different haplotypes of B-*atp6*-GSV occurred independently, indicating that 5′-GGGCGGGGG-3′ and 5′-GGGGGGCGGGA-3′ are the sites prone to chimerism, which was closely associated with the origin of the gametophytic CMS gene. The frequent insertion or deletion of VS segments is the same as the recombination site of nuclear DNA. The high support rate of B-*atp6*-GSV (Fig. 3) and the low support rate in phylogeny tree of GSV (Fig. 5) supported this view. The high support rate of the B-*atp6*-GSV, may be due to the abundant variation sites and long enough sequences, so that the phylogenetic tree can be established more accurately. Due to lack of rich enough variation sites in GSV section, the phylogenetic tree could not reflect the true genetic relationship of different species for the low support rate.

Until now, based on the high homologous sequences in GenBank of NCBI database, eighteen various haplotypes of B-*atp6*-GVS structure including two novel haplotypes (i.e. BH1 and BH3) detected in this study, have been summarized for the ML analysis [36]. Among them, the populations carrying the BH1, BH2 and BH3 formed different clade with a high support rate of 100%, which means their independent occurrence, and doesn't happen randomly. Finding the *B-atp6*-GVS unified chimeric site is very important to explore the molecular mechanism of gametophytic sterility gene production, and understand the mechanism of chimerism, so as to advance the knowledge about the genetic and evolutionary potential principle of chimerism. Only with enough haplotypes, we can find relatively consistent chimeric sites. The haplotypes in this paper is the most haplotype of known chimeric structure. By exploring the chimeric mechanism of sterile genes, we may be able to understand the reasons for the frequent recombination of mtDNA, which might promote the creation of original CMS and understand the mtDNA well.

All these haplotypes show the uniform chimeric trait. GGGCGGGGG……GGGGGCGGG is a similar 123,456,789……987,654,321 unified transposable substructure. The abundant variation sites in the VS region should be attributed to the recombination repair mechanism, which contributed to gain insight into the molecular mechanism of mtDNA chimera formation. Terminal inverted repeats characteristically flank each end of DNA transposons [59, 60]. Transposons are an important source of genetic novelty [61].

### Sequences shared by mitochondrial and nuclear genomes

B*-atp6* is identical to the corresponding sequence of the rice mitochondrial gene *atp6*. The nuclear gene *atp6* is located on chromosome 1 of the ‘Nipponbare’ nuclear genome (AP014957.1) as a highly conserved region [62, 63]. The gene *atp6* also have be found in chromosome 6 of Minghui 63 nuclear genome, chromosome 6 of Shuhui498 for two times, chromosome 1 of *Oryza sativa Japonica* and Zhenshan 97, and chromosome 11, respectively [64–66]. This study confirmed that the VS sequence (176 bp) contains about five insertion or deletion, and more than 30 single nucleotide polymorphism. The first 85 bases of COXII are conserved among all different species, which is shared by the mitochondrial and nuclear genomes. The first 34 bases of GSV are identical to the corresponding sequence of COXII. COXII introns are reported to undergo frequent horizontal transfer [67]. The sequences shared by the mitochondrial and nuclear genomes are responsible for reproductive barriers in rice [68]. Additional research is needed into whether the sequences shared by the mitochondrial and nuclear genomes are associated with the origin of CMS genes and chimeric events.

### Evolution of GSV

The different components of GSV differ in their effect. Pollen of CMS-HL aborts at the binucleate stage, while CMS-BT pollen aborts at the trinucleate stage. Furthermore, pollen abortion occurs at a later stage in the CMS-Lead line than in the CMS-BT line. All three CMS lines show gametophytic sterility [43, 44]. The basal nodes of the phylogenetic tree derived from GSV sequence data cannot reflect the development and evolution of the pollen phenotype. H2 in the top of the phylogenetic tree of GSV, and the H1 is closer to the base. In the process of creating the original CMS source, the parent material initially seed setting normally, the seed setting rate decreased gradually with the breeding crossing, finally the CMS line was bred. It seem as the relationship between H1 and H2 was reflected on the phylogenetic tree of GSV. However, under normal circumstances, we think plant do not allow itself to evolve to extinct. These contradictions reflect the complexity of the evolution of plant mtDNA. The YJ population harboring H3 haplotype shows normal seed set through natural pollination. The GSV haplotype W11 is carried by the *Oryza meridionalis* grown in Australia, the GSV haplotype W21 is carried by the *Oryza barthii* grown in Nigeria, and W34 is carried by *Oryza nivara-*Ura Wee grown in Sri-Lawka, while the GSV haplotype W15 and W29 are carried by the *Oryza nivara* grown in India, and W42 and W46 are carried by *O. rufipogon* grown in Laos and Comodia, respectively [34]. All these materials belong to *Oryza* AA genome. Despite the abundant variation sites in GSV, the close relationship between *O. niwara* and *O. rufipogon* can not be reflected by the ML tree [69] with a relatively low support rate, which should be attributed to the short sequence in GSV with only about 200 bp. On the other hand, at present, their unclear sources also restricted the origin research of gametophytic CMS gene in wild rice.

## Conclusions

In this work, the B*-atp6-orfH79, orfH79* and *atp6* in 427 individuals of seventeen *O. rufipogon* populations in China were amplified using* s*pecific primers through PCR, demonstrating that the gametophytic CMS genes carried by *O. rufipogon* are distributed in transitional geological and ecological niches. Two novel detected haplotypes (i.e. BH1 and BH3) of B*-atp6-orfH79* provided original CMS sources for rice breeding. The sequence characteristics of B*-atp6-orfH79* and ML analysis indicated the chimerise occur independently with consistent chimeric sites, which might help to explore the origin of rice gametophytic CMS genes in *O. rufipogon*. Uniform chimeric traits in the rice *atp6* gene accompany the gametophytic CMS genes. GSV is always accompanied by B-*atp6* in the natural population of *O. rufipogon*.

## Materials and methods

### Plant materials collected in China

A total of 427 individuals with seventeen populations, were identified as *O. rufipogon* by Xuemei Zhang with the assistance of Yating Liu and relevant experts according to its morphological identification, which were collected in from Dongxiang of Jiangxi Province to Sanya of Hainan Province since July 2013 to November 2015 (Fig. 1 and Table 6), representing the geographical distribution of this species in China. Their growing environment and plant appearance of seventeen *Oryza rufipogon* populations were presented in Additional file 2: Figure S1. Samples were randomly collected from individuals separated by at least 5 m to prevent collection of multiple samples from a single genet, except for the NN and YJ populations that the young and healthy leaves of all surviving individuals were sampled, because of the extremely endangered. The collected young and healthy leaves were immediately desiccated in silica gel for DNA extraction.

### DNA extraction

Total genomic DNA was extracted from the desiccated leaf tissue using the cetyltrimethylammonium bromide extraction method established by Doyle & Doyle (1987) [70], and an ammonium acetate wash for additional purification [71]. The DNA was dissolved in 1 × Tris–EDTA buffer (10 mmol/L Tris–HCl, 1 mmol/L EDTA, pH 8.0) to a final concentration of 20–40 ng/µL.

### PCR amplification and sequencing

Specific primers for GSV and B-*atp6*-GSV [8] were used to detect the origin of the gametophytic sterility gene and variation in the sequence structure of B-*atp6*-GSV. Primers for *atp6* amplification were tested on each of the 427 individuals as a positive control for the gametophytic CMS gene. The PCR mixture (total volume 25 µL) contained 2.5 µL of 10 × PCR buffer, 2.5 µL MgCl_2_ (25 mM), 2.0 µL dNTPs mixture (2.5 mM), 0.3 µL each primer (10 µM), 0.2 µL Taq polymerase (5 U/µL) (Sangon Biotech, Shanghai, China), 1 µL template DNA (~ 20–40 ng genomic DNA), and 16.2 µL distilled deionized water. The PCR amplifications were performed on a GeneAmp PCR System 9700 thermal cycler (Perkin Elmer, Foster, CA, USA) with the following protocol: initial denaturation at 95 °C for 4 min, followed by 30 cycles of 95 °C for 1 min, 50–56 °C for 1.5 min, 72 °C for 2 min, and final elongation at 72 °C for 5 min. Amplification products were visualized by 1% agarose gel electrophoresis. If an entire population yielded amplified products, 15 individuals per population were randomly selected for sequencing to evaluate their sequence variations. If a population comprised less than 15 individuals, each individual of the population was sequenced. The PCR products were analyzed and sequenced using an ABI 3700 automated sequencer (Applied Biosystems, Waltham, MA, USA) with the assistance from Sangon Biotech Co., Ltd (Shanghai, China). Contiguous nucleotide sequences were edited using SeqMan in the LaserGene package (DNA Star, Inc., Madison, WI, USA). Consensus sequences were aligned using ClustalX [72] and then adjusted manually.

### Data analysis

The amount of variation among populations within regions and within populations was calculated by means of a hierarchical AMOVA framework [73] using Arlequin 3.0 [74]. Significance was tested using a non-parametric permutation procedure with 1000 permutations. The ML tree was constructed based on evolution distance from the multiple alignment of GSV and B-*atp6*-GSV sequences by PhyML 3.0 software using the maximum likelihood method [75]. The geographical distribution of the mitochondrial haplotypes was mapped by using ArcMap 9.1 (ESRI, Redlands, CA, USA). The BLASTX tool translated the nucleotide sequences into amino acid sequences, and the sequence identities were calculated by the Global Alignment. Additionally, the nucleotide BLAST was used to search for homologous sequences with default parameters in GenBank of NCBI database (https://www.ncbi.nlm.nih.gov).

## Supplementary Information

**Additional file 1:** **Table S1.** 18 Haplotype of B-*atp6*-*orfH79*,and their accession number and relevant population information.

**Additional file 2:** **Fig. S1.** Growing environment and plant appearance of 17 Oryza rufipogonpopulations including BH, DX, FC, GP, GZ, HK, HZ, LB, NHNC, NN, PS, TL, WN, XZ, YJ, YLand QH, that distribute in from northeastern of Jiangxi Province to southeastern of HainanProvince, China.

**Additional file 3:** **TableS2.** Seed setting rate of each individuals collected from TL population located inGuangdong province. **Table S3. **Seed setting rateof each individuals collected from PS population also located in Guangdongprovince. **Table S4. **Seed setting rate of sampled individuals collectedfrom GZ population in Guangdong province.

## Data Availability

The collected plant materials have been deposited in specimen room of The State Key Laboratory of Conservation and Utilization of Bio-resources in Yunnan for the public exhibitions and preservation. All data generated or analyzed during this study are included in this published article. The relevant sequences are available in the GenBank of NCBI database with the accession numbers from KY856719 to KY856721.

## Abbreviations

CMS: Cytoplasmic male sterility
mtDNA: Mitochondrial DNA
CMS-WA: Wild Abortive type CMS
CMS-BT: Boro II type CMS
CMS-HL: HongLian type CMS
PCR: Polymerase chain reaction
IRRI: International Rice Research Institute
GSV: *orf**H**79* And its variants
B-*atp6*-GSV: B-*atp6-orfH79* and its variants
DS: Downstream of the B-*atp6*
VS: Variable sequence
CMS-Dian1: Dian-1 type CMS
CMS-Lead: Lead type CMS
CMS-Liao: Liao type CMS
COXII: Cytochrome oxidase subunit II
EDTA: Ethylene diamine tetraacetic acid
BLAST: Basic Local Alignment Search Tool
AMOVA: Analysis of molecular variation
ML: Maximum likelihood
NCBI: National Center for Biotechnology Information
