# Supplementary material for: Investigation of B-atp6-orfH79 distributing in Chinese populations of Oryza rufipogon and analysis of its chimeric structure
Source: BMC Plant Biol. 2023 Feb 7;23:81. doi: 10.1186/s12870-023-04082-5 (PMC9903446; doi:10.1186/s12870-023-04082-5)
Supplement: Supplementary file 1 — Additional file 1: Table S1. 18 Haplotype of B-atp6-orfH79,and their accession number and relevant population information. [file 12870_2023_4082_MOESM1_ESM.doc]

**Additional file 1: Table S1.** 18 Haplotype of B-*atp6*-*orfH79*, and their accession number and relevant population information

| Haplotype | BH1 | BH2 | BH3 | BH4 | BH5 | BH6 |
| --- | --- | --- | --- | --- | --- | --- |
| Accession No. Population  Species | KY856719  PS, GZ  *O. rufipogon* | KY856720; LR794118.1  TL; HK; CMS-HL  *O. rufipogon* | KY856721  YJ  *O. rufipogon* | LR794108.1  CMS-BT  *O. rufipogon*  *O. sativa*  *O. bathii* | LR794109.1  *O. rufipogon*  *O. sativa* | LR794110.1  *O. rufipogon* |
| Haplotype | BH7 | BH8 | BH9 | BH10 | BH11 | BH12 |
| Accession No. Population  Species | LR794111.1  CMS-Lead  *O. rufipogon*  *O. sativa* | LR794112.1  *O. rufipogon* | LR794113.1  *O. rufipogon*  *O. sativa* | LR794114.1  *O. sativa* | LR794115.1  *O. rufipogon* | LR794116.1  *O. rufipogon* |
| Haplotype | BH13 | BH14 | BH15 | BH16 | BH17 | BH18 |
| Accession No.  Population  Species | LR794117.1  *O. rufipogon* | LR794119.1  *O. rufipogon* | LR7941120.1  *O. rufipogon* | LR794121.1  *O. rufipogon* | LR794122.1  *O. rufipogon* | LR794123.1  *O. rufipogon* |
