# Supplementary material for: Investigation of B-atp6-orfH79 distributing in Chinese populations of Oryza rufipogon and analysis of its chimeric structure
Source: BMC Plant Biol. 2023 Feb 7;23:81. doi: 10.1186/s12870-023-04082-5 (PMC9903446; doi:10.1186/s12870-023-04082-5)
Supplement: Supplementary file 2 — Additional file 2: Fig. S1. Growing environment and plant appearance of 17 Oryza rufipogonpopulations including BH, DX, FC, GP, GZ, HK, HZ, LB, NHNC, NN, PS, TL, WN, XZ, YJ, YLand QH, that distribute in from northeastern of Jiangxi Province to southeastern of HainanProvince, China. [file 12870_2023_4082_MOESM2_ESM.pdf]

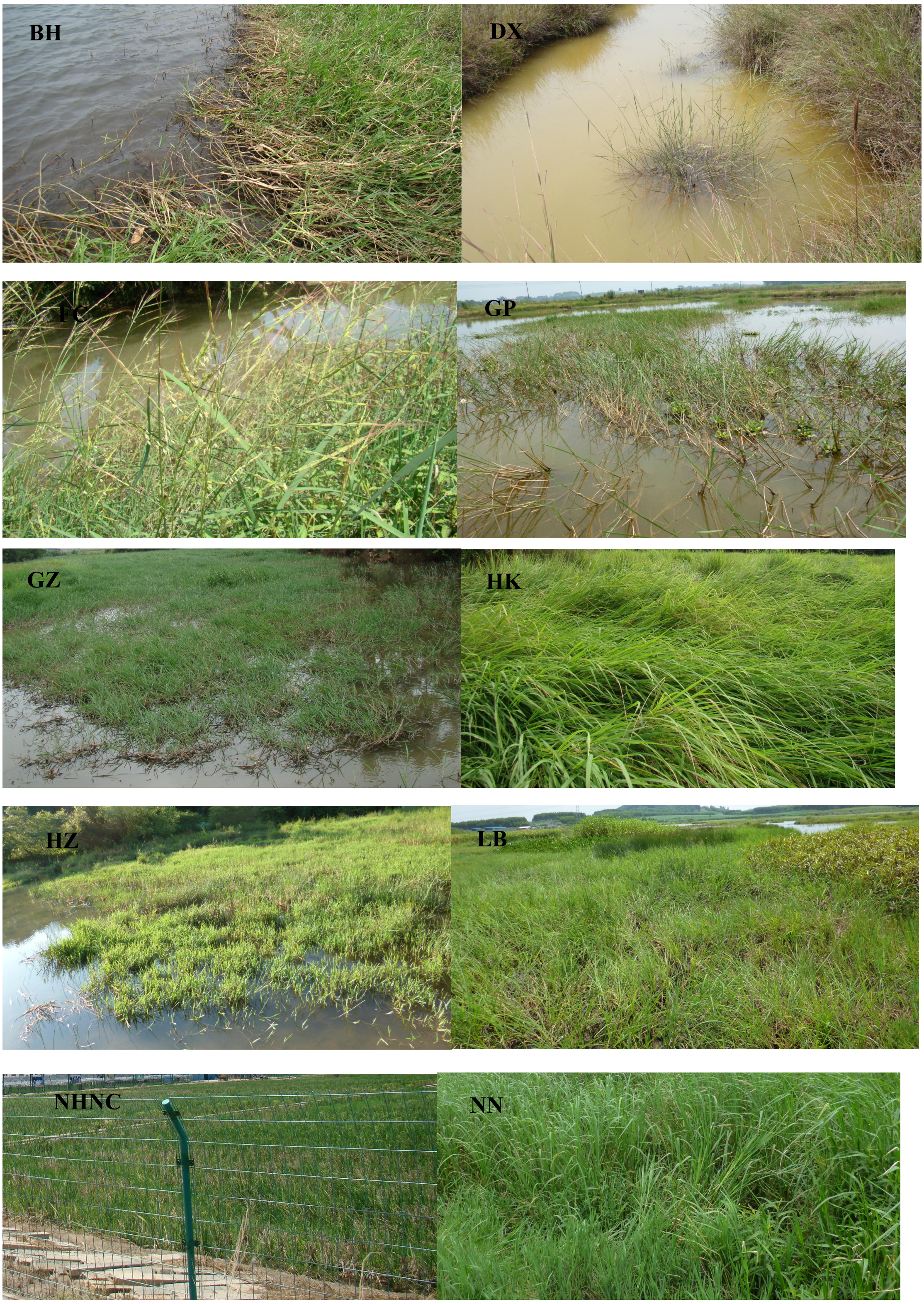
BH
DX
FC
GP
GZ
HK
HZ
LB
NHNC
NN

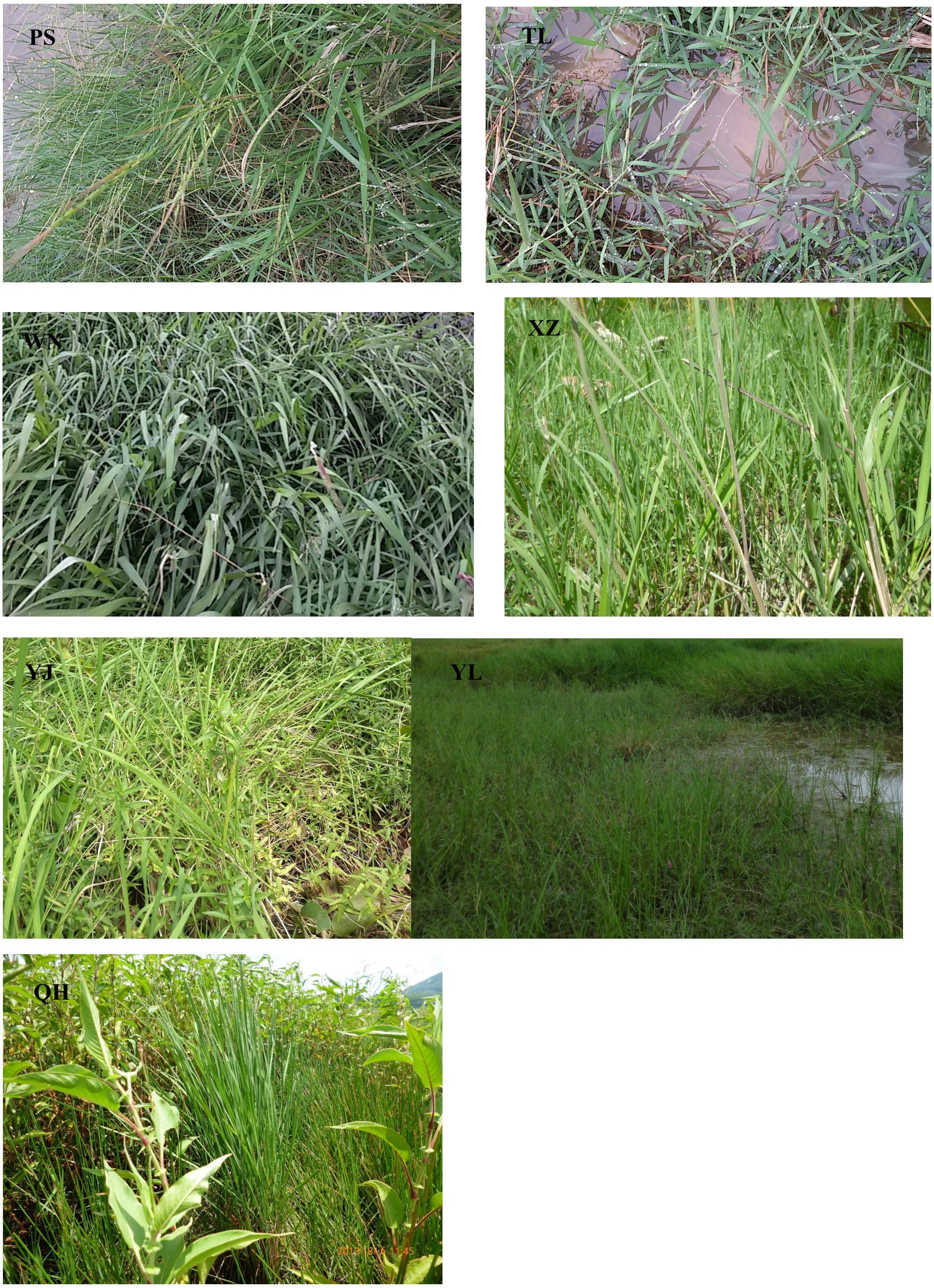

Additional file 2: Fig. S1. Growing environment and plant appearance of 17 *Oryza rufipogon* populations including BH, DX, FC, GP, GZ, HK, HZ, LB, NHNC, NN, PS, TL, WN, XZ, YJ, YL and QH, that distribute in from northeastern of Jiangxi Province to southeastern of Hainan Province, China.
