# Supplementary material for: Investigation of B-atp6-orfH79 distributing in Chinese populations of Oryza rufipogon and analysis of its chimeric structure
Source: BMC Plant Biol. 2023 Feb 7;23:81. doi: 10.1186/s12870-023-04082-5 (PMC9903446; doi:10.1186/s12870-023-04082-5)
Supplement: Supplementary file 3 — Additional file 3: TableS2. Seed setting rate of each individuals collected from TL population located inGuangdong province. Table S3. Seed setting rateof each individuals collected from PS population also located in Guangdongprovince. Table S4. Seed setting rate of sampled individuals collectedfrom GZ population in Guangdong province. [file 12870_2023_4082_MOESM3_ESM.docx]

**Additional File 3: Table S2:** Seed setting rate of each individuals collected from TL population located in Guangdong province.

| Individual code | Grain/Spike | Seed-setting /Spike | Kernel set | Individual code | Grain/Spike | Seed-setting /Spike | Kernel set |
| --- | --- | --- | --- | --- | --- | --- | --- |
| 1 | 28 | 7 | 0.250 | 15 | 25 | 0 | 0.000 |
| 2 | 10 | 0 | 0.000 | 16 | 35 | 0 | 0.000 |
| 2 | 14 | 1 | 0.071 | 17 | 8 | 3 | 0.375 |
| 3 | 24 | 3 | 0.125 | 17 | 19 | 0 | 0.000 |
| 4 | 38 | 1 | 0.026 | 18 | 28 | 0 | 0.000 |
| 5 | 42 | 1 | 0.024 | 19 | 8 | 2 | 0.250 |
| 6 | 34 | 0 | 0.000 | 20 | 17 | 6 | 0.353 |
| 7 | 54 | 9 | 0.167 | 21 | 9 | 0 | 0.000 |
| 8 | 58 | 0 | 0.000 | 22 | 33 | 0 | 0.000 |
| 9 | 54 | 2 | 0.037 | 22 | 46 | 0 | 0.000 |
| 10 | 37 | 0 | 0.000 | 23 | 13 | 1 | 0.077 |
| 10 | 32 | 0 | 0.000 | 24 | 13 | 4 | 0.308 |
| 11 | 43 | 0 | 0.000 | 25 | 7 | 0 | 0.000 |
| 12 | 7 | 0 | 0.000 | 26 | 32 | 0 | 0.000 |
| 13 | 38 | 0 | 0.000 | 26 | 58 | 0 | 0.000 |
| 14 | 12 | 0 | 0.000 | Total | 876 | 40 | 0.046 |

Note: the same code indicates that the rice has two or more panicles.

**Additional File 3: Table S3:** Seed setting rate of each individuals collected from PS population also located in Guangdong province.

| Individual code | Grain/Spike | Seed-setting/Spike | Kernel set | Individual code | Grain/Spike | Seed-setting/Spike | Kernel set |
| --- | --- | --- | --- | --- | --- | --- | --- |
| 1 | 44 | 21 | 0.477 | 14 | 39 | 26 | 0.667 |
| 2 | 25 | 6 | 0.240 | 15 | 37 | 0 | 0.000 |
| 3 | 28 | 19 | 0.679 | 15 | 50 | 2 | 0.040 |
| 4 | 26 | 16 | 0.615 | 16 | 31 | 16 | 0.516 |
| 5 | 28 | 10 | 0.357 | 17 | 14 | 8 | 0.571 |
| 6 | 40 | 28 | 0.700 | 19 | 18 | 2 | 0.111 |
| 7 | 66 | 2 | 0.030 | 20 | 21 | 3 | 0.143 |
| 8 | 42 | 0 | 0.000 | 21 | 33 | 10 | 0.303 |
| 9 | 48 | 2 | 0.042 | 23 | 50 | 1 | 0.020 |
| 10 | 53 | 3 | 0.057 | 24 | 45 | 25 | 0.556 |
| 11 | 61 | 19 | 0.311 | 24 | 41 | 17 | 0.415 |
| 12 | 31 | 24 | 0.774 | 25 | 38 | 11 | 0.289 |
| 12 | 61 | 45 | 0.738 | 25 | 51 | 21 | 0.412 |
| 13 | 28 | 10 | 0.357 | 25 | 44 | 18 | 0.409 |
| 13 | 21 | 9 | 0.429 | Total | 1114 | 374 | 0.336 |

Note: the same code indicates that the rice has two or more panicles.

**Additional File 3: Table S4:** Seed setting rate of sampled individuals collected from GZ population in Guangdong province.

| Individual code | Grain/Spike | Seed-setting/Spike | Kernel set | Individual code | Grain/Spike | Seed-setting/Spike | Kernel set |
| --- | --- | --- | --- | --- | --- | --- | --- |
| 1 | 228 | 3 | 0.013 | 16 | 25 | 14 | 0.560 |
| 2 | 29 | 2 | 0.069 | 17 | 34 | 0 | 0.000 |
| 3 | 19 | 5 | 0.263 | 18 | 29 | 15 | 0.517 |
| 4 | 72 | 6 | 0.083 | 19 | 42 | 14 | 0.333 |
| 5 | 21 | 0 | 0.000 | 20 | 27 | 10 | 0.370 |
| 6 | 33 | 5 | 0.152 | 21 | 28 | 0 | 0.000 |
| 7 | 34 | 14 | 0.412 | 22 | 44 | 1 | 0.0227 |
| 8 | 28 | 6 | 0.214 | 23 | 37 | 0 | 0.000 |
| 9 | 27 | 2 | 0.074 | 24 | 35 | 11 | 0.314 |
| 10 | 28 | 3 | 0.107 | 25 | 28 | 2 | 0.071 |
| 11 | 36 | 3 | 0.083 | 26 | 29 | 2 | 0.069 |
| 12 | 44 | 24 | 0.545 | 27 | 27 | 16 | 0.593 |
| 13 | 48 | 0 | 0.000 | 28 | 25 | 0 | 0.000 |
| 14 | 27 | 11 | 0.407 | 29 | 23 | 4 | 0.174 |
| 15 | 19 | 13 | 0.684 | 30 | 54 | 6 | 0.111 |
|  |  |  |  | Total | 1180 | 192 | 0.163 |

Note: the same code indicates that the rice has two or more panicles.
